# The integration of multidisciplinary approaches revealed PTGES3 as a novel drug target for breast cancer treatment

**DOI:** 10.1186/s12967-024-04899-0

**Published:** 2024-01-20

**Authors:** Qinan Yin, Haodi Ma, Yirui Dong, Shunshun Zhang, Junxiang Wang, Jing Liang, Longfei Mao, Li Zeng, Xin Xiong, Xingang Chen, Jingjing Wang, Xuewei Zheng

**Affiliations:** 1 Precision Medicine Laboratory, School of Medical Technology and Engineering, Henan University of Science and Technology, Luoyang, China; 2 School of Mathematics and Statistics, Henan University of Science and Technology, Luoyang, China; 3 The First Affiliated Hospital of Henan University of Science and Technology, Luoyang, China; 4 College of Basic Medicine and Forensic Medicine, Henan University of Science and Technology, Luoyang, China; 5 Department of Pathology, The First Affiliated Hospital, Jiangxi Medical College, Nanchang University, Nanchang, China

**Keywords:** Breast cancer, Prognostic signature, *PTGES3*, Drug target

## Abstract

**Background:**

The main challenge in personalized treatment of breast cancer (BC) is how to integrate massive amounts of computing resources and data. This study aimed to identify a novel molecular target that might be effective for BC prognosis and for targeted therapy by using network-based multidisciplinary approaches.

**Methods:**

Differentially expressed genes (DEGs) were first identified based on ESTIMATE analysis. A risk model in the TCGA-BRCA cohort was constructed using the risk score of six DEGs and validated in external and clinical in-house cohorts. Subsequently, independent prognostic factors in the internal and external cohorts were evaluated. Cell viability CCK-8 and wound healing assays were performed after *PTGES3* siRNA was transiently transfected into the BC cell lines. Drug prediction and molecular docking between PTGES3 and drugs were further analyzed. Cell viability and PTGES3 expression in two BC cell lines after drug treatment were also investigated.

**Results:**

A novel six-gene signature (including *APOOL*, *BNIP3*, *F2RL2*, *HINT3*, *PTGES3* and *RTN3*) was used to establish a prognostic risk stratification model. The risk score was an independent prognostic factor that was more accurate than clinicopathological risk factors alone in predicting overall survival (OS) in BC patients. A high risk score favored tumor stage/grade but not OS. *PTGES3* had the highest hazard ratio among the six genes in the signature, and its mRNA and protein levels significantly increased in BC cell lines. *PTGES3* knockdown significantly inhibited BC cell proliferation and migration. Three drugs (gedunin, genistein and diethylstilbestrol) were confirmed to target PTGES3, and genistein and diethylstilbestrol demonstrated stronger binding affinities than did gedunin. Genistein and diethylstilbestrol significantly inhibited BC cell proliferation and reduced the protein and mRNA levels of PTGES3.

**Conclusions:**

*PTGES3* was found to be a novel drug target in a robust six-gene prognostic signature that may serve as a potential therapeutic strategy for BC.

**Supplementary Information:**

The online version contains supplementary material available at 10.1186/s12967-024-04899-0.

## Introduction

Breast cancer (BC) is the most common malignancy in women and one of the three most common cancers worldwide, along with lung and colon cancer [1–3]. In 2020, BC was the most commonly diagnosed cancer in females after lung cancer, with an estimated 2.3 million new cases (11.7%), and the death rate reached 6.9% [4]. The main risk factors, including old age, overweight or obesity, tobacco exposure, early menarche, late first-term pregnancy, high breast density and family history of BC, are vital influencing factors for the development of BC [5, 6]. Four molecular subtypes of BC have been characterized: estrogen and progesterone receptor-positive (LuminalA), estrogen, progesterone and HER-2 positive (LuminalB), and HER-2 positive and triple negative BC (TNBC) [7].

The pathogenic mechanisms of BC are diverse, and the typical mechanism is metabolic reprogramming [2]. Profiting from the development of microarray and high-throughput sequencing technology, researchers can identify thousands of cancer-related genes and have innovative insights into their potential molecular mechanisms to apply them to fundamental research to benefit patients [8]. In addition, circRNAs are increasingly used to probe potentially novel biomarkers related to cancer diagnosis, treatment and prognosis [9, 10]. Researchers have shown that mutations in BC-related genes, including BRCA1, BRCA2, ATM and PTEN, can be used as biomarkers to promote personalized treatment of BC patients [11–13]. Unfortunately, the available clinical treatment strategies for BC patients are limited, and the efficacy of these treatments is unsatisfactory. Hence, identification of the original targets is urgently needed to provide new treatment strategies for patients with BC. A prognostic evaluation based on expression profiling and clinical information is critical for making appropriate treatment decisions in BC patients [14]. Therefore, we developed a prognostic model for BC patients to predict overall survival, as this model could provide a basis for the clinical treatment of BC patients in the future.

In this study, a six-gene prognostic risk model was first constructed based on The Cancer Genome Atlas (TCGA) database and then validated using the GSE86166 and METABRIC cohorts. Then, the hub genes were identified in the risk model, and molecular docking analysis was performed to further explore the relationships between the hub genes and potential drugs. Finally, molecular experiments were performed to validate the above results in BC cell lines (Additional file 1: Fig. S1).

## Materials and methods

### Data collection and analysis of immune and stromal scores

The RNA-seq data of BC patients, including 594 samples, were downloaded from the TCGA database (http://portal.gdc.cancer.gov/). Clinical information was extracted from the University of California–Santa Cruz (UCSC) Xena browser (https://xenabrowser.net/datapages/). The TCGA-BRCA data were preprocessed as follows: (1) only one tumor sample was selected; (2) a sample without a specific molecular subtype of BC was excluded; (3) a sample without clinical data and overall survival (OS) < 1 day was excluded; and (4) a gene with a TransPer Kilobase of exon Model per Million mapped reads (TPM) = 0 in 80% of the patients was excluded. Two NCBI Gene Expression Omnibus (GEO) datasets and their clinical information were downloaded from the GEO website (http://www.ncbi.nlm.nih.gov/geo/), the accession numbers of which are GSE86166 (n = 366) and GSE21653 (n = 266). The GEO sets were preprocessed with the following criteria: (1) gene probes were transformed to the human gene SYMBOL, and probes matching multiple genes were removed; if several probes matched one gene, the mean value was selected as the expression profile of the gene; (2) the expression of the gene missing in 80% of the patients was excluded; and (3) the gene was normalized based on the methods described in a previous study (15).

### Identification of differentially expressed genes (DEGs)

The immune score (proportion of immune ingredients), stromal score (proportion of stromal ingredients) and ESTIMATEScore (sum of the above two scores) of each BC sample were calculated using the ESTIMATE package in R software (version: 4.2.0) [16].

The median was set as the cutoff value for the ESTIMATE, immune and stromal scores [17]. Patient samples were divided into two groups based on the average value, namely, the high-score group and the low-score group. The R package limma was used to identify DEGs under the filtering conditions as follows: log (fold change) >|± 2|, with *p* < 0.001. The overlapping DEGs among the three groups mentioned above were identified using a Venn diagram and used for subsequent analysis. A volcano plot was drawn using the ggplot2 R package.

### LASSO regularization and development of the risk score

LASSO can be used in biomarker screening for survival analysis when combined with the Cox model [18]. The analysis was performed using the R package glmnet. Univariate Cox regression analysis was used to calculate regression coefficients, and genes with *p* < 0.05 were used to develop the risk score. Moreover, the survival and survminer R packages were used to compare OS between different groups by Kaplan‒Meier analysis with the log-rank test.

The regression coefficients were subsequently used to calculate the risk score with the following formula: risk score = (exprgene1 * coefficientgene1) + (exprgene2 * coefficientgene2) + … + (exprgenen * coefficientgenen). The BC patients were ultimately stratified into high-risk and low-risk groups based on the median risk score. The survivalROC R package was used to conduct time‐dependent ROC curve analyses to evaluate the predictive power of the gene signature. The risk stratification model was validated using the GSE86166 (n = 366) and METABRIC (n = 1258) cohorts.

Decision curve analysis (DCA) was used to evaluate the net benefit of each gene, and the nomogram was constructed with the R language using the ggDCA package. The immune infiltration of BC patients in the TCGA-BRCA cohort was evaluated using the ssGSEA algorithm and the GSVA R package. A heatmap of immune cells in each sample and different group was generated using the pheatmap R package.

### Functional enrichment analysis and identification of potential therapeutic targets

Gene Ontology (GO) enrichment analysis was performed using the clusterProfiler R package. Kyoto Encyclopedia of Genes and Genomes (KEGG) analyses were performed using the online KOBAS 3.0 database (http://kobas.cbi.pku.edu.cn). All the results were visualized using the ggplot2 package. The FDR method was used to adjust for multiple comparisons, with *p* < 0.05. Protein‒protein interaction (PPI) analysis was performed using GENEMANIA (https://genemania.org/). The potential drugs were identified using the open-source website tool DGIdb (https://dgidb.org/).

### Molecular docking

The protein structure of PTGES3 (PDB ID: 1EJF [19]) was obtained from the RCSB Protein Data Bank. To ensure its suitability for further analysis, the structure underwent several preparatory steps using Schrödinger 2021 software. First, the Protein Preparation Wizard module was used to eliminate crystallographic water molecules, rectify side chains with missing atoms, add hydrogen atoms, and assign protonation states and partial charges using the OPLS4 force field [20]. Subsequently, the protein structure was subjected to minimization until the root-mean-square deviation (RMSD) of the nonhydrogen atoms reached a value of less than 0.3 Å.

Gedunin, genistein, and diethylstilbestrol were prepared for subsequent docking studies. Using the LigPrep module of the Schrödinger 2021 molecular modeling package, these compounds were processed to add hydrogen atoms, convert 2D structures to 3D, generate stereoisomers, and determine the ionization state at pH 7.0 ± 2.0 with Epik [21]. To facilitate the docking process, a receptor grid was generated based on the prepared receptor structure. Finally, the compounds Gedunin, genistein, and diethylstilbestrol were docked to the receptor using the Glide XP protocol.

### Human BC specimens

Twelve BC patients who did not undergo neoadjuvant therapy were collected from the First Affiliated Hospital of Nanchang University, and the mRNA expression of six genes was detected in tumor tissues using qRT‒PCR. The study protocol was approved by the Medical Research Ethics Committee of the First Affiliated Hospital of Nanchang University [Grant no. (2023) CDYFYYLK (04-036)]. All the experiments were performed in compliance with the relevant regulations, and all the patients provided written informed consent.

### Cell culture and transfection

The human nonmalignant breast cell line MCF-10A and two human BC cell lines, MDA-MB-231 and MCF-7, were obtained from Procell (Procell Life Science & Technology Co., Ltd.). MCF-10A cells were cultured in Dulbecco′s modified Eagle′s medium/Ham′s F12 nutrient medium (DMEM/F12; Procell, China) supplemented with 5% horse serum (HS; Procell, China), 20 ng/ml EGF, 0.5 μg/ml hydrocortisone, 10 μg/ml insulin, 1% nonessential amino acids, and 1% penicillin and streptomycin. MDA-MB-231 cells were cultured in Dulbecco’s modified Eagle’s medium (DMEM; Procell, China) supplemented with 10% fetal bovine serum (FBS; Procell, China) and 1% penicillin and streptomycin. MCF-7 cells were maintained in minimum essential medium (MEM; Procell, China) supplemented with 0.01 mg/ml insulin, 10% FBS and 1% penicillin and streptomycin. All cell lines were kept at 37 °C and 5% CO_2_ in a humidified atmosphere.

Small interfering RNAs (siRNAs) targeting the PTGES3 coding sequence (5′-GUCAGUGUUCCAGGUGUAUTT-3′) was obtained from GenePharma (China) according to previous methods [22]. The siRNA sequence was transfected into cells (70% confluence) using jetPRIME transfection reagent (Polyplus, France) in strict accordance with the manufacturer's instructions. After incubating for 48 h, the cells were collected and used to detect protein and mRNA expression.

### Scratch wound healing assay

The transfected BC cells were seeded into 6-well plates and incubated at 37 °C in 5% CO_2_ until 100% confluence was reached. Thereafter, the monolayer was scraped with a 200 μL pipette. After removing the cell debris, the cells were cultured under normal conditions. Images were taken at 0 h and 48 h, which show the relative distance between two edges.

### Cell viability assay

BC cells were seeded in a 96-well plate (3000 cells/well), and genistein and diethylstilbestrol were subsequently added to the cell culture media at concentrations of 100 μM, 50 μM, 20 μM, 10 μM and 5 μM at 37 °C in a 5% CO_2_ environment. The transfected BC cells were collected at 24 h and 48 h before the cell viability assay. Cell viability was measured by a Cell Counting Kit-8 (Beyotime, Shanghai, China) according to the manufacturer's instructions. The absorbance was measured at 450 nm by a microplate reader (Bio-Rad, CA, USA). Moreover, the expression level of PTGES3 was determined after 48 h of drug intervention (50 μM).

### qRT‒PCR

TransZol reagent purchased from TransGen (Beijing, China) was used to extract total RNA from the cell lines and tissues. Three micrograms of total RNA were subjected to reverse transcription using HiScript II RT SuperMix for qPCR (+ gDNA wiper) (Vazyme, Nanjing, China) reagent according to the manufacturer's instructions. Next, 2 × SYBR Green qPCR Master Mix II (SEVEN, Beijing, China) was used to perform qRT‒PCR. Finally, the relative expression levels of mRNA were calculated by the 2^−△△Ct^ method, and GAPDH was used as an internal control. The cell experiments were carried out using three biological replicates. The results were further normalized to the GADPH expression. The primers used are listed in Additional file 2: Table S1.

### Western blot and immunohistochemistry

Cells were lysed using RIPA lysis buffer (Solarbio, Beijing, China) containing 1% protease inhibitor (PMSF) to extract total protein, and the protein concentration was determined using a BCA protein concentration assay kit (Beyotime, Shanghai, China). Subsequently, the proteins were separated via 12% SDS‒PAGE and transferred to polyvinylidene difluoride (PVDF) membranes (Millipore, MA, USA). After blocking in 5% nonfat goat milk powder for 2 h, the membrane and primary antibody (anti-PTGES3: 1:5000 dilution; Proteintech, Wuhan, China; Cat# 67,736–1-Ig; anti-β-actin: 1:1000 dilution; Cell Signaling Technology, MA, USA; Cat# 8H10D10) were incubated overnight at 4 °C, and horseradish peroxidase (HRP)-conjugated secondary antibody goat anti-mouse IgG (H + L) (1:5,000 dilution; DY60203; Diyibio, Shanghai, China) was added for 2 h at room temperature. Finally, the protein bands were visualized using a chemiluminescence instrument. Western blot analysis was performed with ImageJ software. The Human Protein Atlas (HPA) database (https://www.proteinatlas.org/) was also used to verify protein expression levels.

### Statistical analysis

All the statistical analyses were performed using the R language. Nonparametric tests (Wilcoxon rank-sum test for independent groups and Wilcoxon signed-rank test for paired groups) were used to compare the median values of two sets of continuous variables. *p* < 0.05 was considered to indicate statistical significance, and all the statistical tests were two-sided.

## Results

### Identification of the hub genes in the TCGA-BRCA cohort

**Fig. 1:**
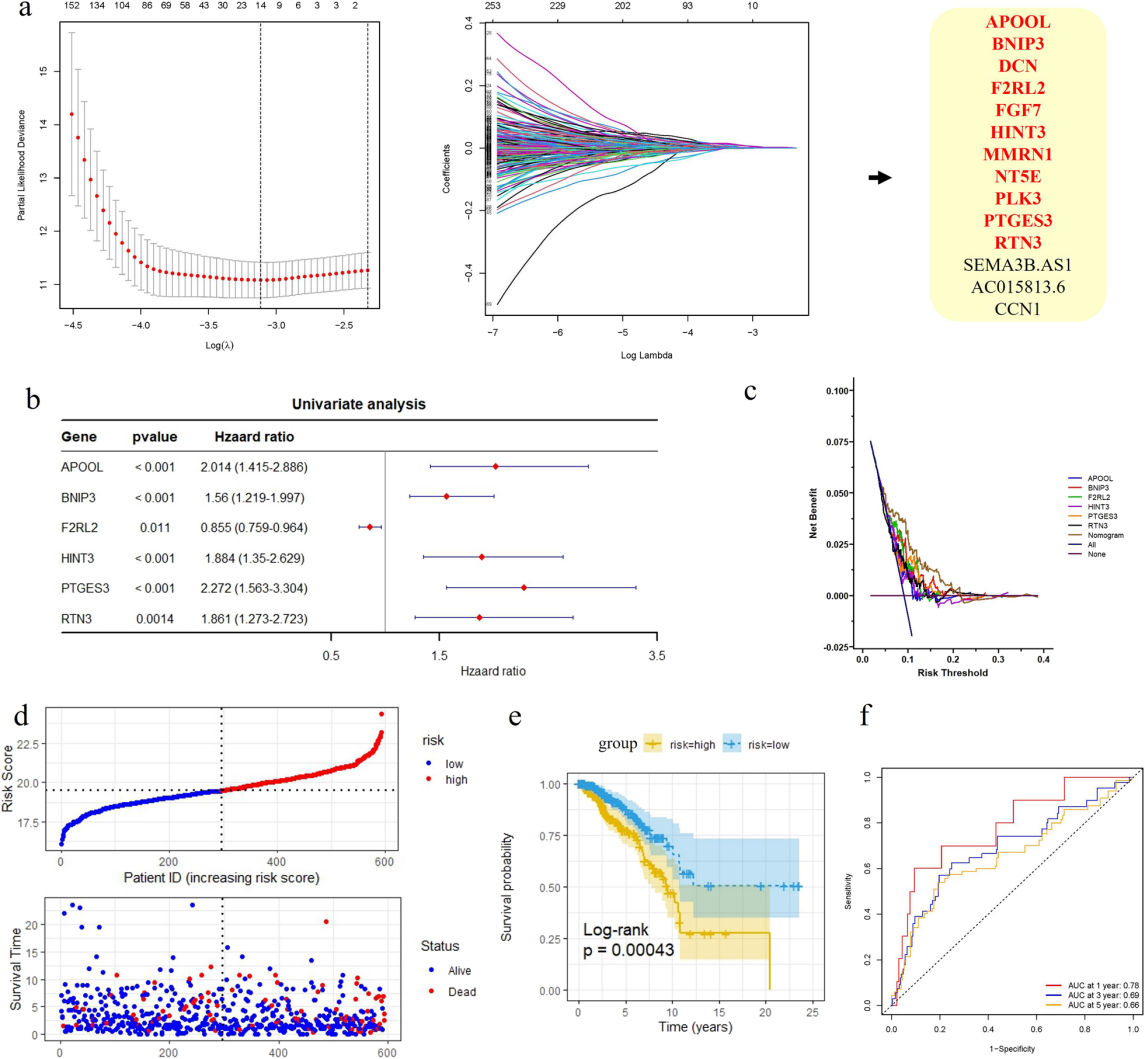
Prognostic analysis of the six-gene signature model in the TCGA-BRCA cohort. **a** Tuning parameter selection by tenfold cross-validation in the LASSO model and trajectory changes of each independent variable. The partial likelihood deviance was plotted against log (Lambda/λ), and λ was the tuning parameter. The Y-axis shows LASSO coefficients and the X-axis is − log (lambda); **b** Univariate Cox regression analysis result of six genes; **c** DCA analysis; **d** The distributions of overall survival status, overall survival and risk score in the TCGA-BRCA cohort; Hierarchical clustering of six core genes between low- and high-risk groups. Red, up-regulated; Blue, down-regulated; **e** Kaplan–Meier survival curve based on the six-gene signature; **f** ROC curve of the 6-gene signature for 1-year, 3-year and 5-year survival in the training set

The ImmuneScore, StromalScore, and ESTIMATEScore were first calculated for the TCGA-BRCA cohort. K‒M curves revealed the correlation of immune and stromal proportions with OS, indicating that the immune score was positively correlated with OS (log-rank *p* = 0.01; Additional file 1: Fig. S2a). However, neither the StromalScore (log-rank *p* = 0.25; Additional file 1: Fig. S2b) nor the ESTIMATEScore (log-rank *p* = 0.77; Additional file 1: Fig. S2c) was significantly correlated with OS. We further identified the DEGs in the samples, and the volcano plot shows upregulated and downregulated DEGs compared with those in the low-loading group (Figures S2a-c). A total of 2018 overlapping DEGs (Additional file 1: Fig. S2d, Additional file 2: Table S2-S4), including 996 upregulated and 1022 downregulated DEGs, were obtained and used for further analysis. The 2018 DEGs were subjected to dimensional reduction analysis via LASSO regression analysis. A total of 14 hub genes were identified through LASSO-Cox analysis (minimum error rate λ = 0.044) and were significantly associated with OS (*p* < 0.05) (Fig. 1a). Unfortunately, three hub genes, *AC015813.6*, *CCN1* and *SEMA3B-AS1*, were identified are unique to the TCGA database; thus, they were excluded from the following analysis.

Univariate Cox proportional hazard regression suggested that five candidate genes, *APOOL*, *BNIP3*, *HINT3*, *PTGES3* and *RTN3,* were not associated with BC prognosis (Fig. 1b), except for *F2RL2*. The DCA analysis indicated that the predictive ability of the nomogram model was greater than that of the other components at the high-risk threshold (Fig. 1c), suggesting that the nomogram model has the highest predictive ability. K‒M analysis revealed that high expression levels of five candidate genes (*APOOL, BNIP3, F2RL2, HINT3, PTGES3 and RTN3*) were significantly positively associated with poor prognosis (Additional file 1: Fig. S3). Moreover, a high *F2RL2* expression level was positively associated with a good prognosis (Additional file 1: Fig. S3a).

### Construction and validation of the risk stratification model in in/external cohorts

Thus, we further constructed a prognostic risk model by calculating the risk score as follows: risk score = 0.700*exp^APOOL^ + 0.445*exp^BNIP3^—0.156*exp^F2RL2^ + 0.633*exp^HINT3^ + 0.821*exp^PTGES3+^ 0.621*exp^RTN3^. Based on the median risk score (7.364; Additional file 2: Table S7), BC patients were categorized into high-risk or low-risk groups (Fig. 1d). There was a greater mortality rate in the high-risk group than in the low-risk group (Fig. 1e). The OS of patients in the high-risk group was significantly lower than that of patients in the low-risk group (*p* < 0.001). The predictive performance of the risk score for OS was evaluated by time-dependent ROC curves, and the area under the curve (AUC) reached 0.78 at 1 year, 0.69 at 3 years, and 0.66 at 5 years (Fig. 1f).

**Fig. 2:**
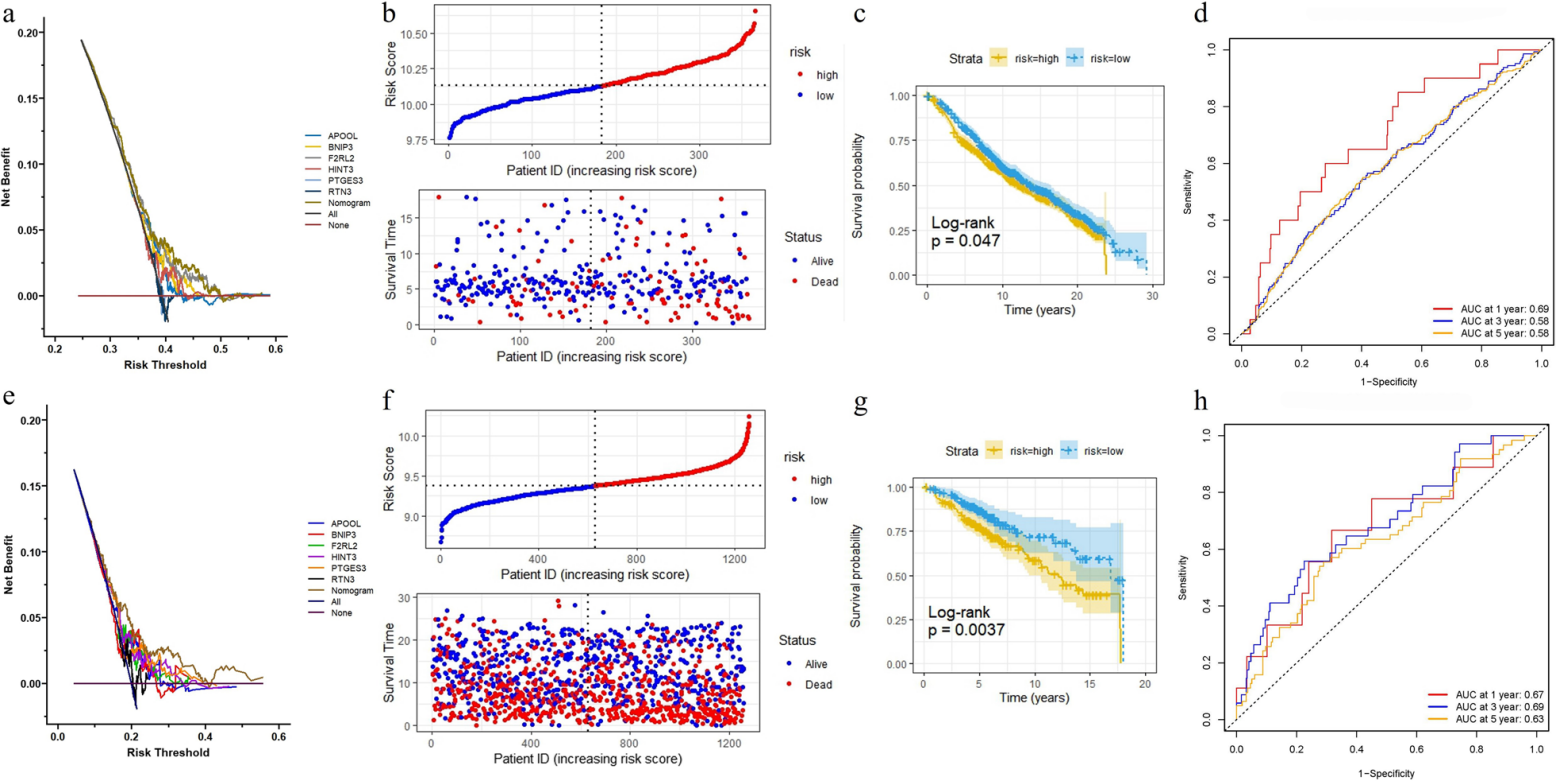
Validation of the risk model basing on six-gene in GEO and METABRIC cohort. **a**, **e** DCA analysis. **b**, **f** The distributions of overall survival status, overall survival and risk score; Hierarchical clustering of six core genes between low- and high-risk groups. Red, up-regulated; Blue, down-regulated. **c**, **g** Kaplan–Meier survival curve based on the six-gene signature. **d**, **h** ROC curve of the 6-gene signature for 1-year, 3-year and 5-year survival

To test the stability and accuracy of the model, the prediction efficiency of our six-gene signature was also calculated in both external validation cohorts (METABRIC and GSE86166). The DCA analysis indicated the same results (Fig. 2a, e) as in the TCGA-BRAC cohort, suggesting that the nomogram model based on six genes is a robust risk model. Patients in both cohorts were also divided into high-risk and low-risk groups, and the median value was calculated using the same formula used for the TCGA-BRCA cohort (Fig. 2b, f). In both external cohorts (Fig. 2c, g), patients in the low-risk score group had longer OS than did those in the high-risk group (METABRIC cohort, log-rank *p* = 0.047; GSE86166 cohort, log-rank *p* = 0.0037). The ROC curve showed that the accuracy of the prognostic six-gene signature for 1-year, 3-year and 5-year survival was 0.69, 0.58 and 0.58, respectively, in the METABRIC cohort (Fig. 2d), and 0.67, 0.69 and 0.63, respectively, in the GSE86166 cohort (Fig. 2e). Therefore, the results indicated that the six-gene prognostic model for predicting the cancer-specific survival of BC patients has good reliability and repeatability.

In addition, functional enrichment analysis revealed that the six novel genes were related mainly to RNA-directed DNA polymerase activity (MF), mitochondrial membrane organization and energy derivation by oxidation of organic compounds (BP), and the organelle outer membrane (CC) (Additional file 1: Fig. S3b, Additional file 2: Table S5). KEGG pathway analysis revealed that *PTGES3* participates in the regulation of the arachidonic acid metabolism pathway and metabolic pathways; *BNIP3* participates in the regulation of legionellosis, mitophagy-animal, autophagy-animal and FoxO signaling pathways; *F2RL2* participates in the regulation of complement and coagulation cascades; and *RTN3* participates in the regulation of Alzheimer disease (Additional file 1: Fig. S3c, Additional file 2: Table S6). The PPI network analysis revealed that 6 genes significantly interacted with 23 genes (Additional file 1: Fig. S3d).

### Independent prognostic value of the six-gene signature

**Fig. 3:**
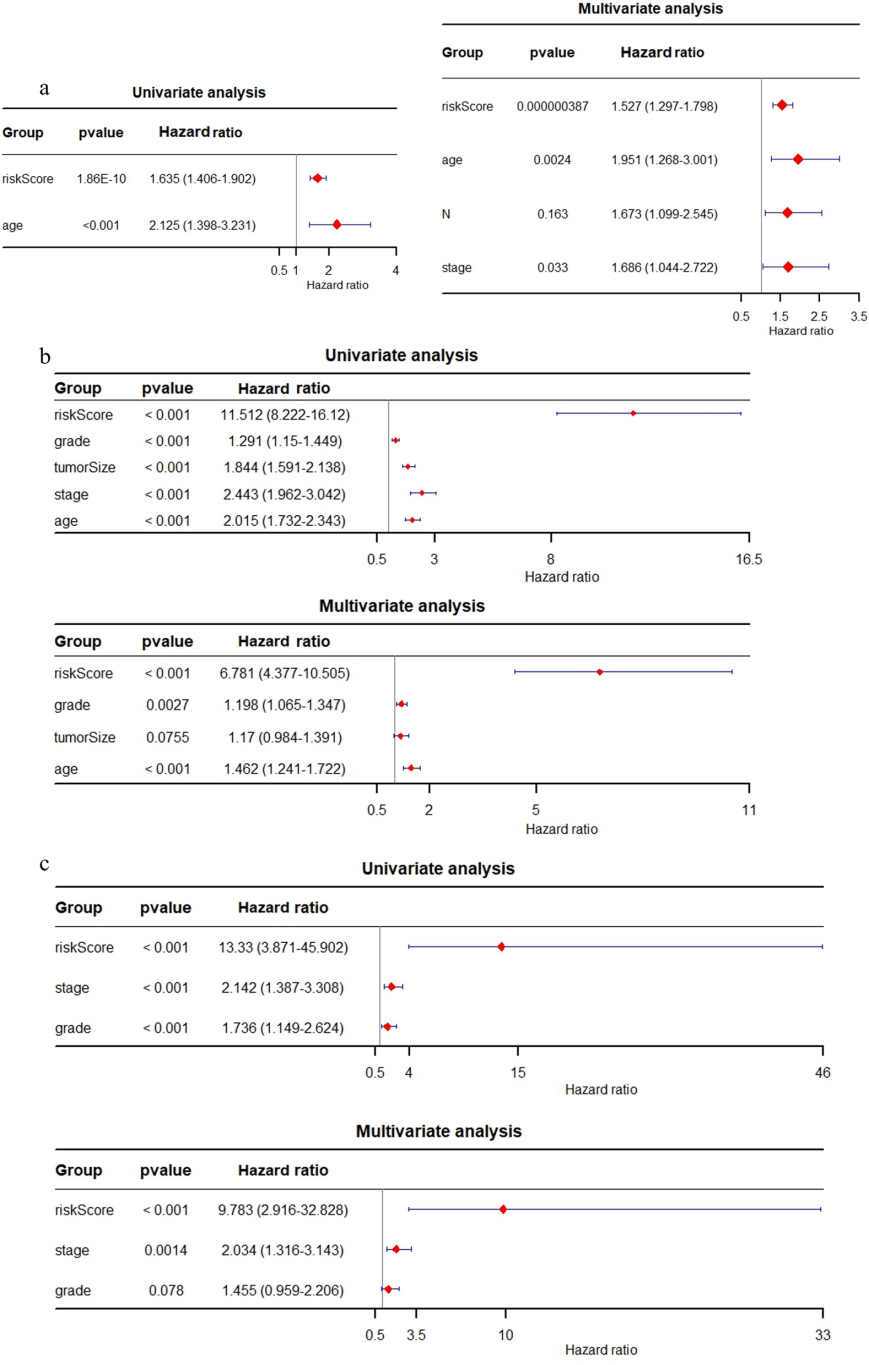
Univariate and multivariate Cox regression analyses of the six-gene signature. **a** Forest plot of the univariate and multivariate Cox regression analyses in TCGA-BRCA cohort. **b** Forest plot of the univariate and multivariate Cox regression analyses in METABRIC cohort. **c** Forest plot of the univariate and multivariate Cox regression analyses in the GSE86166 cohort

Univariate and multivariate Cox regression analyses were carried out among the available variables to determine whether the risk score was an independent prognostic predictor for OS. According to univariate Cox regression analyses, the risk score was significantly associated with OS in the TCGA-BRCA, METABRIC and GSE86166 cohorts (TCGA-BRCA cohort: HR = 1.635, 95% CI 1.406–1.902, *p* = 1.86E-10; METABRIC cohort: HR = 11.512, 95% CI 8.22–16.12, *p* < 0.001; GSE86166 cohort: HR = 13.33, 95% CI 3.871–45.902, *p* < 0.001) (Fig. 3). The risk score still proved to be an independent predictor of OS after correction for other confounding factors according to multivariate Cox regression analysis (TCGA-BRCA cohort: HR = 1.527, 95% CI 1.297–1.798, *p* = 3.87E−7; METABRIC cohort: HR = 6.781 95% CI 4.377–10.505, *p* < 0.001; GSE86166 cohort: HR = 9.783, 95% CI 2.916–32.828, *p* < 0.001) (Fig. 3). In addition, clinicopathological parameters, including tumor grade, tumor size and stage, were identified as independent prognostic factors in both external cohorts (Fig. 3b, c). In summary, the six-gene signature can be considered an independent prognostic indicator of BC.

**Fig. 4:**
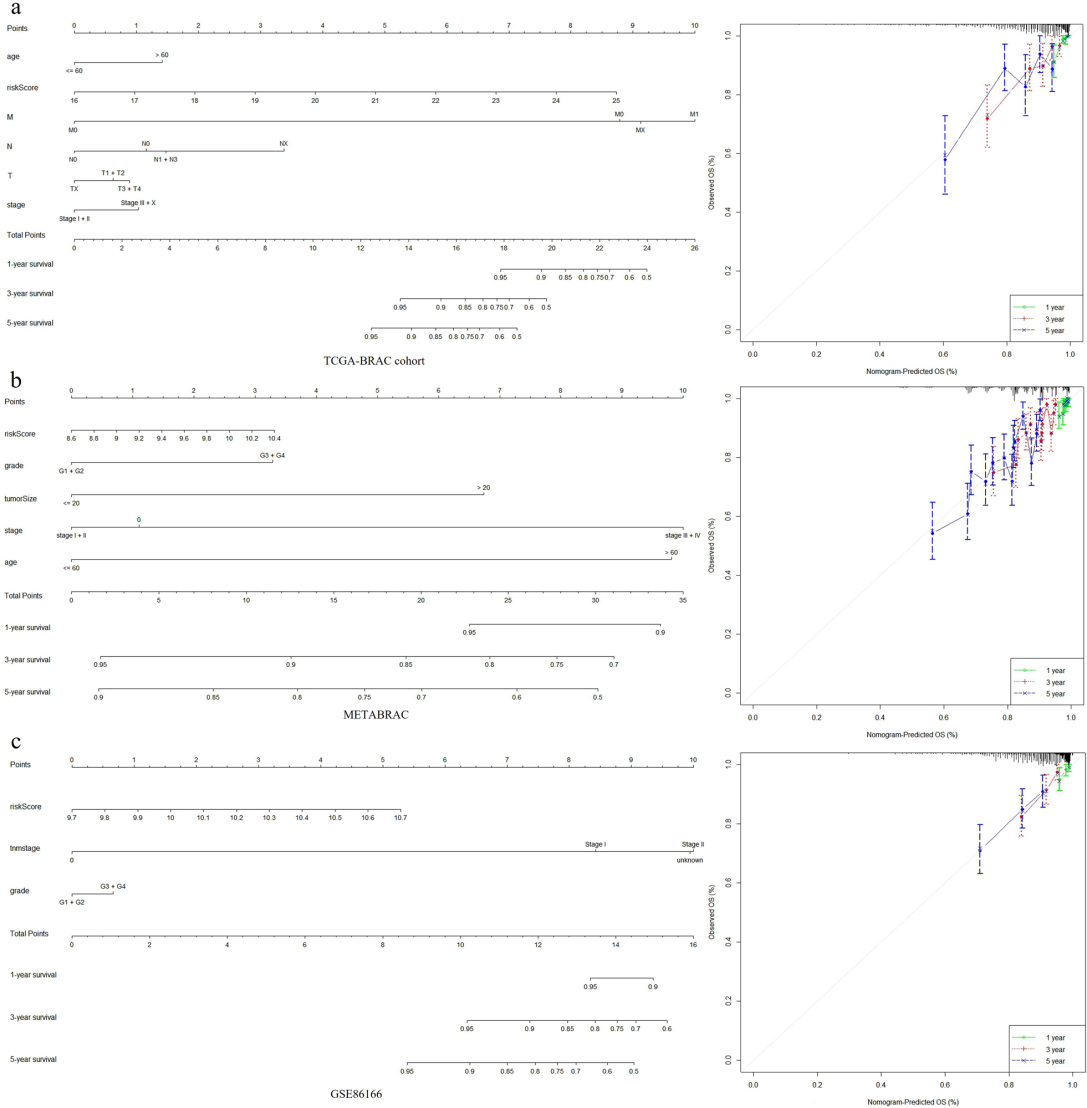
Nomograms for prediction of the outcome of patients with breast cancer. **a** Nomogram developed by integrating the signature risk-score with the clinicopathologic features in the TCGA-BRCA cohort. And calibration curves of nomogram for predicting overall survival at 1-year, 3-year and 5-year in the TCGA-BRCA cohort. **b** Validated by METABRIC cohort. **c** Validated by GSE86166 cohort

All the parameters, including the risk score, were subsequently used to develop a nomogram in the in- and external cohorts. By scoring the predictors, the survival time decreased as the total score increased. The calibration curves of the 1-year, 3-year and 5-year OS probabilities showed good consistency between the nomogram predictions and the results observed in the TCGA-BRCA cohort (Fig. 4a). Moreover, the nomogram and calibration curves for the probabilities of 1-, 3-, and 5-year OS in both external cohorts indicated excellent agreement between the nomogram prediction and observed outcomes (Fig. 4b, c). In summary, the risk score was better able to predict OS in BC patients and could be an independent prognostic factor.

### The baseline characteristics of the BC patients and risk score distributions for clinicopathological parameters in the external cohorts

The high-risk group was significantly associated with older age and advanced TN stage in the TCGA-BRCA cohort (Table 1). For both external cohorts,

**Table 1:** Baseline characteristics of the BRCA patients between the low and high-risk groups in different cohorts

Characteristic	TCGA-BRCA cohort			METABRIC cohort			GSE86166 cohort		
	Low risk	High risk	*p*	Low risk	High risk	*p*	Low risk	High risk	*p*
Age (mean (SD))	56.34(12.78)	59.90(12.72)	0.001	60.78 (12.26)	60.61 (13.70))	0.87			
M (%)			0.492						
M0	268(90.2)	262(88.5)							
M1	4(1.3)	8(2.7)							
MX	25(8.4)	26(8.8)							
N (%)			0.041						
N0	140(47.1)	134(45.3)							
N1	106(35.7)	91(30.7)							
N2	30(10.1)	47(15.9)							
N3	19(6.4)	15(5.1)							
NX	2(0.7)	9(3.0)							
T (%)			0.008						
T1	95(32.0)	57(19.3)							
T2	164(55.2)	194(65.5)							
T3	29(9.8)	31(10.5)							
T4	9(3.0)	13(4.4)							
TX	0(0.0)	1(0.3)							
Grade (%)						< 0.001			0.002
G1				62(10.2)	43(6.6)		25(13.7)	11(6.0)	
G2				272(44.9)	209(32.1)		85(46.4)	63(34.4)	
G3				272(44.9)	400(61.3)		66(36.1)	102(55.7)	
G4							6(3.3)	5(2.7)	
G5							1(0.5)	2(1.1)	
Tumor size (mean (SD))				24.72 (12.85)	27.12 (16.68)	0.005			
Stage (%)			0.143			0.714			0.221
Unknown				0(0.0)	1(0.2)		7(3.8)	3(1.6)	
Stage I	55(18.5)	37(12.5)		212(35.0)	210(32.2)		2(1.1)	2(1.1)	
Stage II	175(58.9)	172(58.1)		341(56.3)	378(58.0)		62(33.9)	49(26.8)	
Stage III	61(20.5)	76(25.7)		50(8.3)	59(9.0)		84(45.9)	92(50.3)	
Stage IV	4(1.3)	6(2.0)		3(0.5)	4(0.6)				
Stage X	2(0.7)	5(1.7)							

The high-risk subgroup in the METABRIC cohort was significantly associated with higher tumor grade and size (Table 1). The high-risk group in the GSE86166 cohort was significantly associated with higher tumor grade (Table 1).

**Fig. 5:**
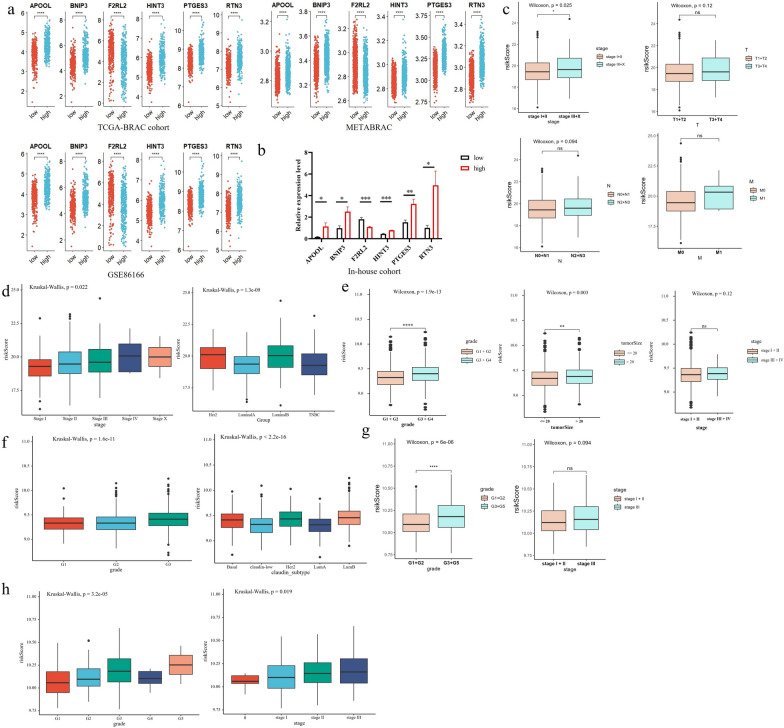
Expression level of six genes and clinicopathological parameters in the risk model. Expression level of 6 genes in TCGA-BRAC, METABRIC, GSE86166 cohorts (**a**) and in-house cohort (**b**). The risk score distribution in early- and advanced- breast cancer in TCGA-BRAC cohort (**c**), METABRIC cohort (**e**) and GSE86166 cohort (**g**). The risk score distribution in different subtypes and pathologic stage in TCGA-BRAC cohort (**d**), METABRIC cohort (**f**), and GSE86166 cohort (**h**); *p < 0.05, **p < 0.01; ***p < 0.005, ****p < 0.001, ns, no significance

Except for the downregulated expression of the prognostic protective gene F2RL2 in the high-risk group, the other five genes were upregulated in the high-risk group compared with the low-risk group in the three cohorts (Fig. 5a). Similar results were further confirmed in the clinical in-house cohort (Fig. 5b). The risk scores were significantly greater (*p* = 0.025) in the advanced-stage tumor cohort (from stage III to stage X) than in the early-stage tumor cohort (from stage I to stage II) in the TCGA-BRCA cohort (Fig. 5c). Interestingly, advanced tumor stage was associated with a high risk score, and the risk score was significantly distributed across multiple BC subtypes in the TCGA-BRCA cohort (Fig. 5d). Accordingly, the risk score was also significantly associated with advanced tumor grade (G3 + G4 in the METABRIC cohort or G3 + G5 in the GSE86166 cohort) and tumor size (> 20 in the METABRIC cohort) in both external cohorts (Fig. 5e, g). Moreover, advanced tumor stage/grade was associated with a high risk score (Fig. 5f, h), and the risk score was also significantly distributed among the BC subtypes in the METABRIC cohort (Fig. 5f). In conclusion, the results showed that the six-gene signature is an accurate prognostic model for BC patients, possesses a high ability to distinguish early and advanced BC, and could be a novel tool for the prediction of malignant tumors.

The infiltration of immune cells was also evaluated using the ssGSEA algorithm in the TCGA-BRAC cohort and in the low- and high-risk groups (Additional file 1: Fig. S4a). A total of 23 of the 27 immune cells exhibited significant differences between the two groups, among which 4 immune cell types increased and 19 immune cell types decreased in the high-risk group compared with the low-risk group (Additional file 1: Fig. S4). In addition, the distribution of immune cells was significantly different between patients with early-stage tumors and patients with advanced-stage tumors (Additional file 1: Fig. S4b).

### PTGES3 was a potential therapeutic target in the six-gene signature

Because the ImmuneScore was positively correlated with OS (Additional file 1: Fig. S2a), the correlation between the ImmuneScore and risk score was calculated (Additional file 1: Fig. S5a). The risk score was negatively correlated with the ImmuneScore; 5 genes (APOOL, BNIP3, HINT3, PTGES3 and RTN3) were positively correlated with the risk score and negatively correlated with the ImmuneScore (Additional file 1: Fig. S5b, c); and F2RL2 was negatively correlated with the risk score and positively correlated with the ImmuneScore (Additional file 1: Fig. S5b, c).

**Fig. 6:**
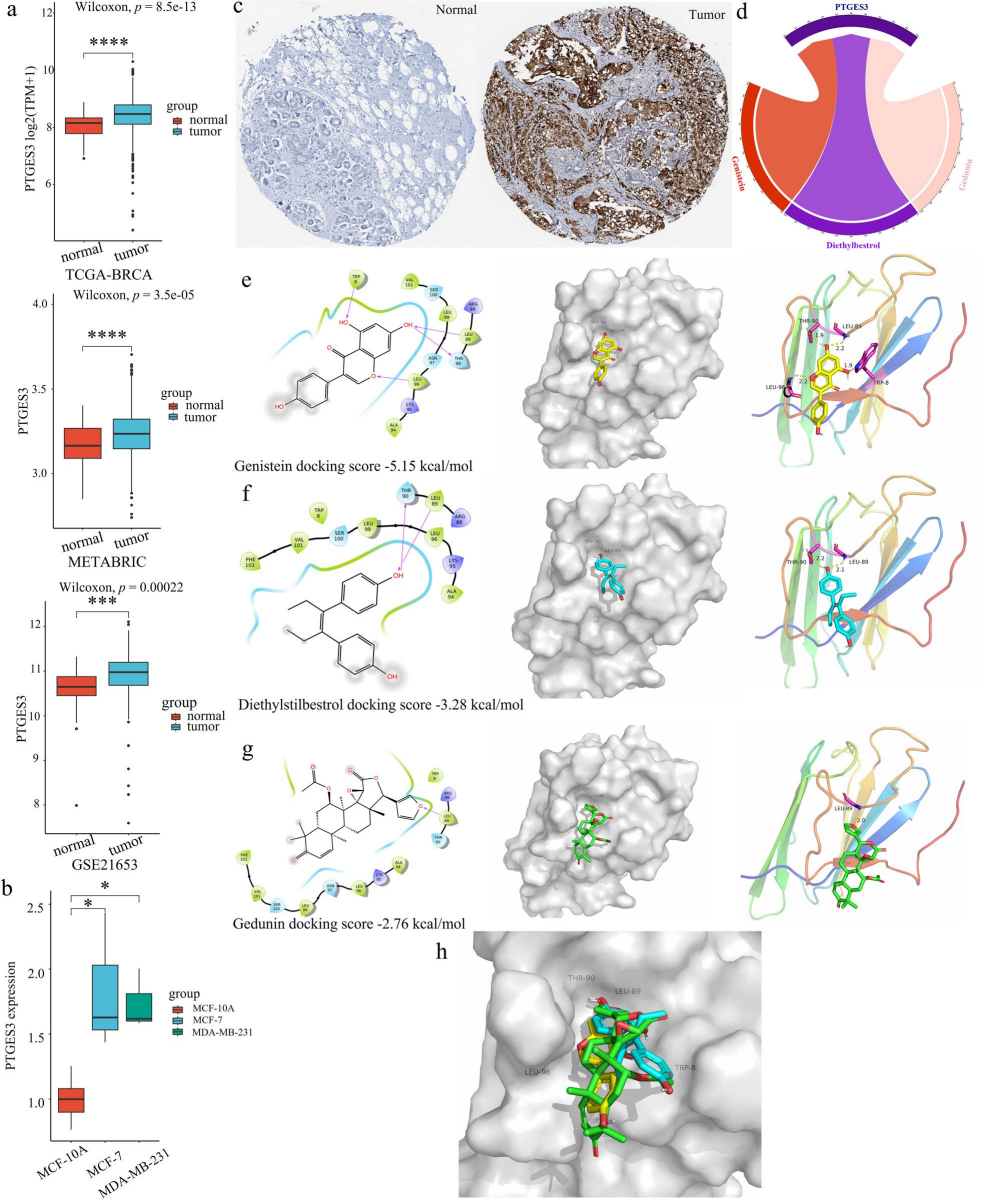
Expression level of PTGES3 in breast cancer and molecular docking analysis. **a** mRNA expression level in three cohorts. **b** The mRNA expression level in breast cancer cell lines; **c** Protein level in breast cancer samples. **d** Potential drugs targeted PTGES3; Genistein (**e**), Diethylstilbestrol (**f**) and Gedunin (**g**) docked into the PTGES3 crystal structure. **h** Molecular docking analysis of three drugs. *p < 0.05, **p < 0.01; ***p < 0.005, ****p < 0.001

**Fig. 7:**
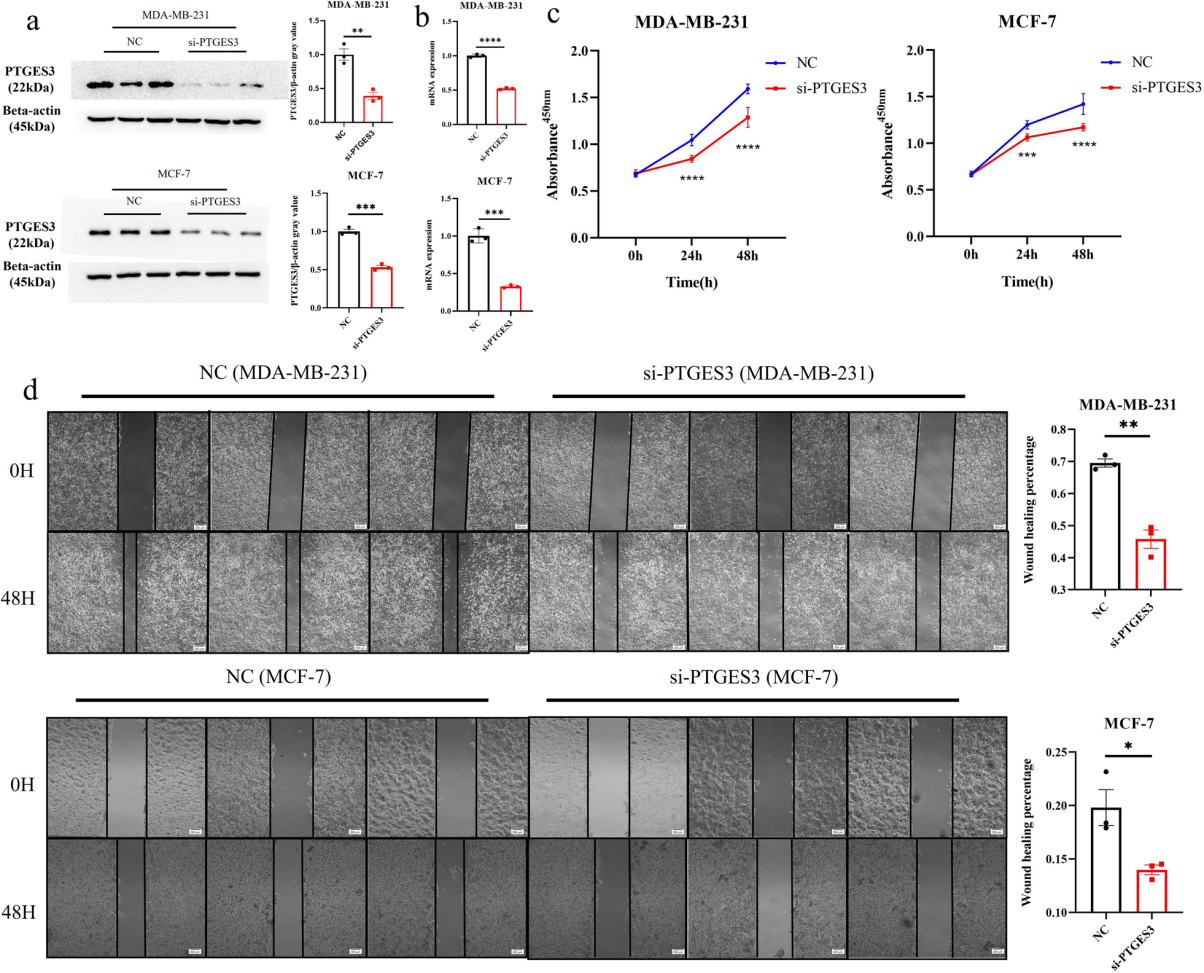
Decreased PTGES3 expression effectively inhibits cell proliferation and migration in breast cancer cell lines. Protein (**a**) and mRNA (**b**) levels of PTGES3 in MDA-MB-231 and MCF-7 cells. **c** Cell viability CCK-8 assay; **d** Wound-healing assay; **p < 0.01; ***p < 0.005, ****p < 0.001. NC, negative control

**Fig. 8:**
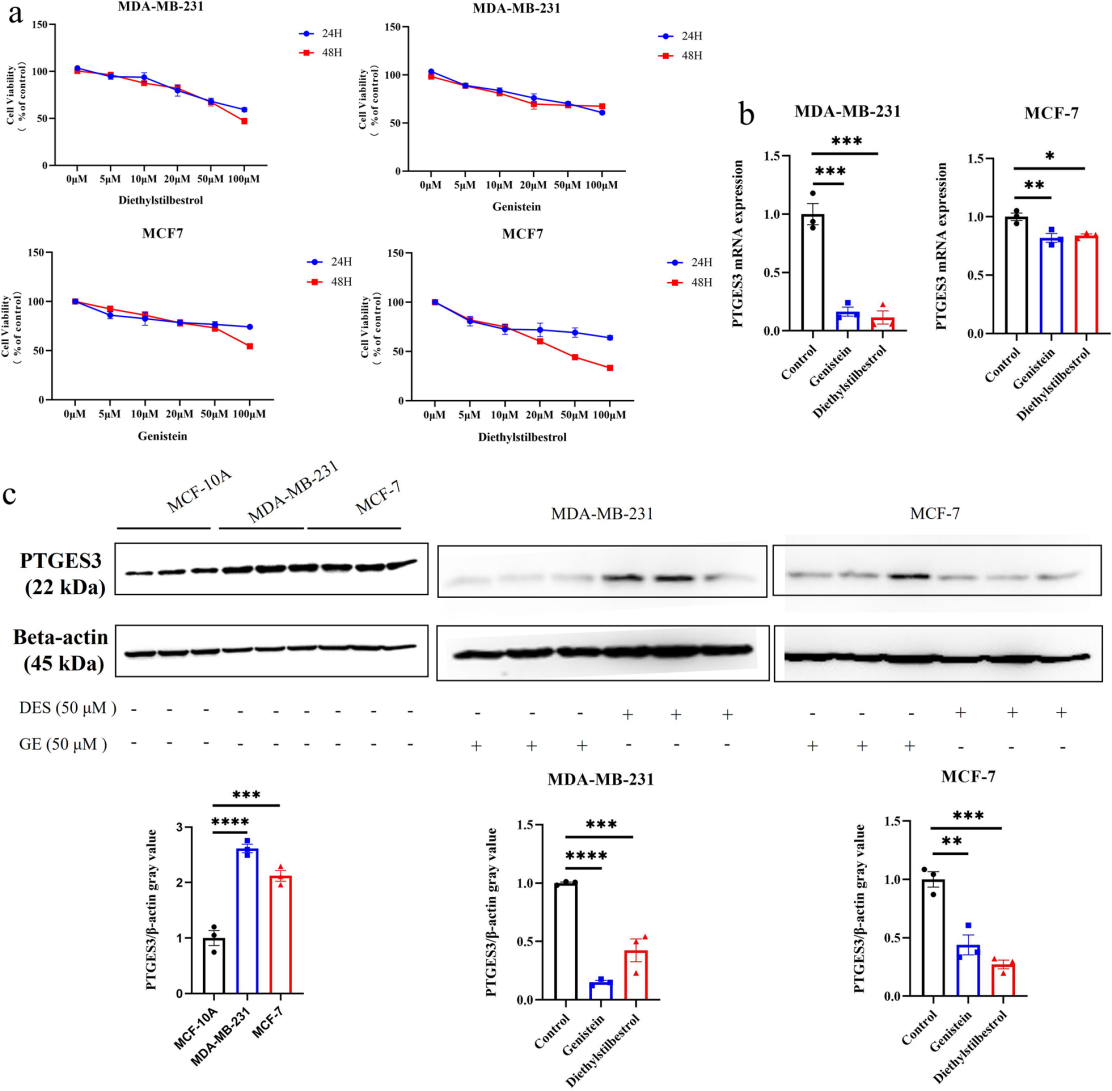
Genistein and diethylstilbestrol inhibited cell viability and suppressed the expression of PTGES3. **a** Effect of genistein and diethylstilbestrol on cell viability measured by CCK-8 assay in breast cancer cell lines; The mRNA expression (**b**) and protein level (**c**) of PTGES3 were decreased after exposure to the genistein and diethylstilbestrol in breast cancer cell lines. **p < 0.01; ***p < 0.005, ****p < 0.001. DES, diethylstilbestrol; GE, genistein

Notably, PTGES3 had the highest HR (Fig. 1b) and highest correlation with the risk score (Additional file 1: Fig. S5b); thus, its mRNA and protein levels were detected. The mRNA expression levels in the TCGA-BRCA, METABRIC and GSE21653 cohorts were greater in the tumor group than in the normal group (Fig. 6a). These results were confirmed in two BC cell lines (MDA-MB-231 and MCF-7) and in MCF-10A cells (Fig. 6b). Moreover, the protein level of PTGES3 was also increased according to immunochemical analysis of the BC samples (Fig. 6c), and increased protein levels were also observed in the two BC cell lines (Fig. 8c). The results showed that *PTGES3* siRNAs significantly decreased PTGES3 expression at the protein and mRNA levels after they were transiently transfected into the two BC cell lines (Fig. 7a, b). *PTGES3* knockdown significantly reduced the viability and migration of both BC cell lines (Fig. 7c, d).

Notably, PTGES3 was a novel potential target of gedunin, genistein and diethylstilbestrol (Fig. 6d). Therefore, molecular docking between the drugs above and PTGES3 was carried out to further clarify the possibility that PTGES3 is a potential target. Gedunin has been confirmed to dock and inactivate PTGES3 [23]. Overall, genistein and diethylstilbestrol demonstrated stronger binding affinities to PTGES3 than did gedunin based on docking scores and interaction analysis (Fig. 6e–h). In terms of docking scores, genistein exhibited the highest score of − 5.15 kcal/mol, followed by diethylstilbestrol, with a score of − 3.28 kcal/mol. Both of these scores surpassed gedunin’s docking score of − 2.76 kcal/mol (Fig. 6e–g). According to the interaction analysis, genistein formed four hydrogen bonds with the amino acids TRP8, LEU89, THR90, and LEU96 (Fig. 6e, h). Diethylstilbestrol formed two hydrogen bond interactions with LEU89 and THR90 (Fig. 6f, h). On the other hand, Gedunin only formed one hydrogen bond with LEU89 (Fig. 6g, h).

Subsequently, the effects of genistein and diethylstilbestrol on BC cell proliferation were investigated. The results indicated that both drugs significantly inhibited the viability of BC cells (Fig. 8a). Both drugs significantly inhibited the expression of PTGES3 at the mRNA and protein levels (Fig. 8b, c), suggesting that they inhibited *PTGES3* expression in BC cells.

## Discussion

As the most common type of malignancy, BC affects approximately 12% of women worldwide [24]. There are many treatment strategies for this disease, including resection, chemotherapy and radiotherapy. These methods are constantly improving, but because of their limited sensitivity and specificity, the prognosis of BC is not ideal [25, 26]. For the prevention, screening, diagnosis and treatment of BC, accurate prognostic biomarkers or signatures that aid in predicting the survival of BC patients are needed.

In this study, we constructed a robust six-gene signature independent of clinical factors to predict the OS of BC patients. Among the prognostic risk genes in the risk model, *APOOL*, *BNIP3*, *HINT3*, *PTGES3* and *RTN3* were associated with a high risk score. In contrast, high expression of the prognostic protective gene *F2RL2* was a protective factor. Studies have indicated that *BNIP3* plays an important role in the regulation of autophagy, metabolic pathways, and metastasis-related processes in different tumor types [27, 28] and can be regulated by *p21* to promote cell cycle arrest in pancreatic cancer [29]. *BNIP3* promotes cell apoptosis in lung cancer [30] and pancreatic cancer [29] and is also a predictive factor for lung adenocarcinoma and small-cell lymphoma in individuals in the autophagy-related biomarker groups, indicating that *BNIP3* is a prognostic marker [30]. In lung adenocarcinoma, *PTGES3* is also an independent poor prognostic biomarker, and a high *PTGES3* expression level is associated with immune invasion in cancer cells [31]. *PTGES3* knockdown significantly inhibited lung tumor growth [22], and these results were also confirmed in our study. *PTGES3* knockdown markedly suppressed BC cell proliferation and migration. KEGG enrichment analysis revealed that *PTGES3* participates in the regulation of the arachidonic acid metabolism pathway, which has been proven to be a new therapeutic target for BC metastasis [32], but its specific molecular mechanism needs to be further explored. A study showed that *RTN3* functions as a new inhibitor and can activate the Chk2/p53 pathway to suppress hepatocellular carcinoma, which provides additional clues for a better understanding of the carcinogenic role of HBV [33]. Many other studies have shown that *F2RL2* is a biomarker for a variety of tumors, such as colon adenocarcinoma and glioma [34, 35]. In BC patients, *F2RL2* is associated with the process by which metastatic BC spreads to bone [36]. *APOOL* and *HINT3* are tumor-related genes that were first discovered in this study as new prognostic markers for BC, but the mechanism of these genes in BC remains to be clarified.

Notably, *PTGES3* was the key gene with the highest hazard ratio and was the target of gedunin, genistein and diethylstilbestrol. Chaitanya [23] demonstrated that Gedunin can bind to the surface of PTGES3 and promote cancer cell apoptosis. Here, similar molecular docking results were confirmed between PTGES3 and Gedunin. Interestingly, genistein and diethylstilbestrol had stronger binding affinities for PTGES3 than did gedunin. Two drugs, for instance, a high dose of genistein, induce BC cell apoptosis [37] and increase the sensitivity of TNBC to antiestrogen therapy [38]. In the present study, genistein and diethylstilbestrol inhibited BC cell growth and decreased PTGES3 expression at the protein and mRNA levels, indicating that the antitumor effects of these drugs were closely related to *PTGES3*. However, the potential underlying mechanisms should be explored in further experiments.

Based on univariate and multivariate Cox proportional risk model analyses, Liang et al. also established a new ferritin decorrelation gene marker to predict the overall survival rate of patients with hepatocellular carcinoma [39]. Wang [40] also developed a prognostic marker that included nine ferroptosis-related genes via the LASSO-Cox regression model and suggested that this gene could be used as a new biomarker to predict the prognosis of BC patients. Moreover, Zheng et al. constructed an 8-gene signature based on the energy metabolism of esophageal carcinoma [41]. In contrast to the findings of previous research, the risk model in this study was accurate in characterizing the immune response and predicting overall survival in patients with BC. In this study, Kaplan‒Meier curves confirmed that all six genes were significantly related to OS, and the AUC of the ROC curve in TCGA, METABRIC and GSE86166 datasets was close to 0.7. The results showed that the six-gene signature had a high AUC and involved fewer genes, which could show strong potential for clinical translation and help in the clinical diagnosis of BC.

Some limitations of this study should also be noted. First, our prognostic model was both constructed and validated with retrospective data from public databases; if additional prospective real-world data are available, its clinical utility can be better verified. Second, our sample lacked clinical follow-up information; therefore, factors such as other health conditions were not considered when determining prognostic biomarkers. Additionally, the results obtained through bioinformatics analysis alone were less convincing and need to be verified by further molecular biology experiments. Finally, further genetic and experimental studies based on a larger sample size and experimental validation are needed.

In summary, the results showed that the novel six-gene signature has prognostic value for additional clinicopathological risk parameters and is more accurate at predicting OS in BC patients than clinicopathological risk factors alone. This risk model might, therefore, provide solid support for patient risk stratification and precise management of BC patients. However, *PTGES3* was confirmed to be a novel drug target that may serve as a therapeutic target for BC.

### Supplementary Information

**Additional file 1: Figure S1.** The flow chart for whole process analysis of this study. **Figure S2.** The Kaplan–Meier basing on the ESTIMATE analysis and Venn diagram of the differentially expressed genes (DEGs). The Kaplan–Meier curve and volcano plot basing on **a** immune score, **b** stromal score and **c** ESTIMATE score; **d** Venn diagram of the up-regulation and down-regulation DEGs. The overlapped DEGs are used for further analysis. **Figure S3.** The Kaplan–Meier and functional analysis. **a** Survival analyses according to the optimal cut-off expression value of each gene in the TCGA-BRCA cohort. All *p* < 0.05; **b** GO enrichment analysis; **c** KEGG analysis; **d** Protein–protein interaction analysis of six genes. **Figure S4.** Single sample gene set enrichment (ssGSEA) analysis in TCGA-BRAC cohort. **a** The expression levels of different immune cells in low- and high-risk groups; **b** The distribution of immune cells; red font represents upregulation and blue font represents downregulation; *p < 0.05, **p < 0.01; ***p < 0.005, ****p < 0.001. **Figure S5.** Correlation analysis for the six genes in TCGA-BRAC cohort. **a** The correlation between riskScore and immunScore. **b** The correlation between riskScore and 6 genes. **c** The correlation between immuneScore and six genes.

**Additional file 2: Table S1.** The primers applied in qRT-PCR. **Table S2.** The DEGs between high and low immune score group. **Table S3.** The DEGs between high and low strimal score group. **Table S4.** The DEGs between high and low ESTIMATE score group. **Table S5.** The GO enrichment analysis of six candidate genes. **Table S6.** The KEGG enrichment analysis of six candidate genes. **Table S7.** The risk model in TCGA-BRCA cohort.

## Data Availability

The datasets used and/or analyzed during the current study are available from the corresponding author on reasonable request.

## References

[1] Harbeck N, Gnant M (2017). Breast cancer. Lancet.

[2] Zheng X, Ma H, Wang J, Huang M, Fu D, Qin L (2022). Energy metabolism pathways in breast cancer progression: the reprogramming, crosstalk, and potential therapeutic targets. Transl Oncol.

[3] Yin Q, Ma H, Bamunuarachchi G, Zheng X, Ma Y (2023). Long non-coding RNAs, cell cycle, and human breast cancer. Hum Gene Ther.

[4] Sung H, Ferlay J, Siegel RL, Laversanne M, Soerjomataram I, Jemal A (2021). Global cancer statistics 2020: GLOBOCAN estimates of incidence and mortality worldwide for 36 cancers in 185 countries. CA Cancer J Clin.

[5] Britt KL, Cuzick J, Phillips KA (2020). Key steps for effective breast cancer prevention. Nat Rev Cancer.

[6] Zhang Y, Zhou Y, Mao F, Yao R, Sun Q (2020). Ki-67 index, progesterone receptor expression, histologic grade and tumor size in predicting breast cancer recurrence risk: a consecutive cohort study. Cancer Commun (Lond).

[7] Goldhirsch A, Wood WC, Coates AS, Gelber RD, Thurlimann B, Senn HJ (2011). Strategies for subtypes–dealing with the diversity of breast cancer: highlights of the St. Gallen International Expert Consensus on the Primary Therapy of Early Breast Cancer 2011. Ann Oncol..

[8] Cui J, Yin Y, Ma Q, Wang G, Olman V, Zhang Y (2015). Comprehensive characterization of the genomic alterations in human gastric cancer. Int J Cancer.

[9] Cancer Genome Atlas Research Network (2014). Comprehensive molecular characterization of gastric adenocarcinoma. Nature..

[10] Zheng X, Ma H, Dong Y, Fang M, Wang J, Xiong X (2023). Immune-related biomarkers predict the prognosis and immune response of breast cancer based on bioinformatic analysis and machine learning. Funct Integr Genomics.

[11] Dorling L, Carvalho S, Allen J, González-Neira A, Luccarini C, Wahlström C (2021). Breast cancer risk genes: association analysis in more than 113,000 women. N Engl J Med.

[12] Shiovitz S, Korde LA (2015). Genetics of breast cancer: a topic in evolution. Ann Oncol.

[13] Tray N, Taff J, Adams S (2019). Therapeutic landscape of metaplastic breast cancer. Cancer Treat Rev.

[14] Rakha EA, Reis-Filho JS, Baehner F, Dabbs DJ, Decker T, Eusebi V (2010). Breast cancer prognostic classification in the molecular era: the role of histological grade. Breast Cancer Res.

[15] Gautier L, Cope L, Bolstad BM, Irizarry RA (2004). affy–analysis of Affymetrix GeneChip data at the probe level. Bioinformatics.

[16] Yoshihara K, Shahmoradgoli M, Martínez E, Vegesna R, Kim H, Torres-Garcia W (2013). Inferring tumour purity and stromal and immune cell admixture from expression data. Nat Commun.

[17] Xiang S, Li J, Shen J, Zhao Y, Wu X, Li M (2021). Identification of prognostic genes in the tumor microenvironment of hepatocellular carcinoma. Front Immunol.

[18] Tibshirani R (1997). The lasso method for variable selection in the Cox model. Stat Med.

[19] Weaver AJ, Sullivan WP, Felts SJ, Owen BA, Toft DO (2000). Crystal structure and activity of human p23, a heat shock protein 90 co-chaperone. J Biol Chem.

[20] Lu C, Wu C, Ghoreishi D, Chen W, Wang L, Damm W (2021). OPLS4: improving force field accuracy on challenging regimes of chemical space. J Chem Theory Comput.

[21] Shelley JC, Cholleti A, Frye LL, Greenwood JR, Timlin MR, Uchimaya M (2007). Epik: a software program for pK a prediction and protonation state generation for drug-like molecules. J Comput Aided Mol Des.

[22] Yu Z, Peng Y, Gao J, Zhou M, Shi L, Zhao F (2023). The p23 co-chaperone is a succinate-activated COX-2 transcription factor in lung adenocarcinoma tumorigenesis. Sci Adv..

[23] Patwardhan CA, Fauq A, Peterson LB, Miller C, Blagg BS, Chadli A (2013). Gedunin inactivates the co-chaperone p23 protein causing cancer cell death by apoptosis. J Biol Chem.

[24] Akram M, Iqbal M, Daniyal M, Khan AU (2017). Awareness and current knowledge of breast cancer. Biol Res.

[25] Curigliano G, Burstein HJ, Winer EP, Gnant M, Dubsky P, Loibl S (2017). De-escalating and escalating treatments for early-stage breast cancer: the St. Gallen international expert consensus conference on the primary therapy of early breast cancer 2017. Ann Oncol..

[26] Shi M, Guo N (2009). MicroRNA expression and its implications for the diagnosis and therapeutic strategies of breast cancer. Cancer Treat Rev.

[27] Evison M, AstraZeneca UKL (2020). The current treatment landscape in the UK for stage III NSCLC. Br J Cancer.

[28] Nollet EA, Cardo-Vila M, Ganguly SS, Tran JD, Schulz VV, Cress A (2020). Androgen receptor-induced integrin α6β1 and Bnip3 promote survival and resistance to PI3K inhibitors in castration-resistant prostate cancer. Oncogene.

[29] Manu KA, Chai TF, Teh JT, Zhu WL, Casey PJ, Wang M (2017). Inhibition of isoprenylcysteine carboxylmethyltransferase induces cell-cycle arrest and apoptosis through p21 and p21-regulated BNIP3 induction in pancreatic cancer. Mol Cancer Ther.

[30] Gorbunova AS, Yapryntseva MA, Denisenko TV, Zhivotovsky B (2020). BNIP3 in lung cancer: to kill or rescue?. Cancers (Basel)..

[31] Gao P, Zou K, Xiao L, Zhou H, Xu X, Zeng Z (2022). High expression of PTGES3 is an independent predictive poor prognostic biomarker and correlates with immune infiltrates in lung adenocarcinoma. Int Immunopharmacol.

[32] Borin TF, Angara K, Rashid MH, Achyut BR, Arbab AS (2017). Arachidonic acid metabolite as a novel therapeutic target in breast cancer metastasis. Int J Mol Sci..

[33] Song S, Shi Y, Wu W, Wu H, Chang L, Peng P (2021). Reticulon 3-mediated Chk2/p53 activation suppresses hepatocellular carcinogenesis and is blocked by hepatitis B virus. Gut.

[34] Lvu W, Fei X, Chen C, Zhang B (2020). In silico identification of the prognostic biomarkers and therapeutic targets associated with cancer stem cell characteristics of glioma. Biosci Rep..

[35] Zhou R, Gao Z, Ju Y (2022). Novel six-gene prognostic signature based on colon adenocarcinoma immune-related genes. BMC Bioinformatics.

[36] Liu S, Song A, Wu Y, Yao S, Wang M, Niu T (2021). Analysis of genomics and immune infiltration patterns of epithelial-mesenchymal transition related to metastatic breast cancer to bone. Transl Oncol.

[37] Chen J, Duan Y, Zhang X, Ye Y, Ge B, Chen J (2015). Genistein induces apoptosis by the inactivation of the IGF-1R/p-Akt signaling pathway in MCF-7 human breast cancer cells. Food Funct.

[38] Donovan MG, Selmin OI, Doetschman TC, Romagnolo DF (2019). Epigenetic activation of BRCA1 by genistein in vivo and triple negative breast cancer cells linked to antagonism toward aryl hydrocarbon receptor. Nutrients..

[39] Liang JY, Wang DS, Lin HC, Chen XX, Yang H, Zheng Y (2020). A novel ferroptosis-related gene signature for overall survival prediction in patients with hepatocellular carcinoma. Int J Biol Sci.

[40] Wang D, Wei G, Ma J, Cheng S, Jia L, Song X (2021). Identification of the prognostic value of ferroptosis-related gene signature in breast cancer patients. BMC Cancer.

[41] Zheng W, Chen C, Yu J, Jin C, Han T (2021). An energy metabolism-based eight-gene signature correlates with the clinical outcome of esophagus carcinoma. BMC Cancer.

