# Supplementary material for: The integration of multidisciplinary approaches revealed PTGES3 as a novel drug target for breast cancer treatment
Source: J Transl Med. 2024 Jan 20;22:84. doi: 10.1186/s12967-024-04899-0 (PMC10800054; doi:10.1186/s12967-024-04899-0)
Supplement: Supplementary file 1 — Additional file 1: Figure S1. The flow chart for whole process analysis of this study. Figure S2. The Kaplan–Meier basing on the ESTIMATE analysis and Venn diagram of the differentially expressed genes (DEGs). The Kaplan–Meier curve and volcano plot basing on a immune score, b stromal score and c ESTIMATE score; d Venn diagram of the up-regulation and down-regulation DEGs. The overlapped DEGs are used for further analysis. Figure S3. The Kaplan–Meier and functional analysis. a Survival analyses according to the optimal cut-off expression value of each gene in the TCGA-BRCA cohort. All p < 0.05; b GO enrichment analysis; c KEGG analysis; d Protein–protein interaction analysis of six genes. Figure S4. Single sample gene set enrichment (ssGSEA) analysis in TCGA-BRAC cohort. a The expression levels of different immune cells in low- and high-risk groups; b The distribution of immune cells; red font represents upregulation and blue font represents downregulation; *p < 0.05, **p < 0.01; ***p < 0.005, ****p < 0.001. Figure S5. Correlation analysis for the six genes in TCGA-BRAC cohort. a The correlation between riskScore and immunScore. b The correlation between riskScore and 6 genes. c The correlation between immuneScore and six genes. [file 12967_2024_4899_MOESM1_ESM.docx]

**Integration of multidisciplinary approaches revealed PTGES3 as** **a novel** **drug target in breast cancer therapeutic strategy**

Qinan Yin^1^, Haodi Ma^1^, Shunshun Zhang^1^, Yirui Dong^1^, Junxiang Wang^2^, Jing Liang^3^, Longfei Mao^4^, Li Zeng^1^, Xingang Chen^1^, Jingjing Wang^1^, Xuewei Zheng^1*^

^1^Precision Medicine Laboratory, School of Medical Technology and Engineering, Henan University of Science and Technology, Luoyang, China.

^2^School of Mathematics and Statistics, Henan University of Science and Technology, Luoyang, China

^3^The First Affiliated Hospital of Henan University of Science and Technology, Luoyang, China

^4^College of Basic Medicine and Forensic Medicine, Henan University of Science and Technology, Luoyang, China

^*^Corresponding author:

Xuewei Zheng

Precision Medicine Laboratory,

School of Medical Technology and Engineering,

Henan University of Science and Technology, Luoyang, China.

Email: [xwzheng0529@163.com](mailto:xwzheng0529@163.com)

**Running Title**: PTGES3 is a novel drug target


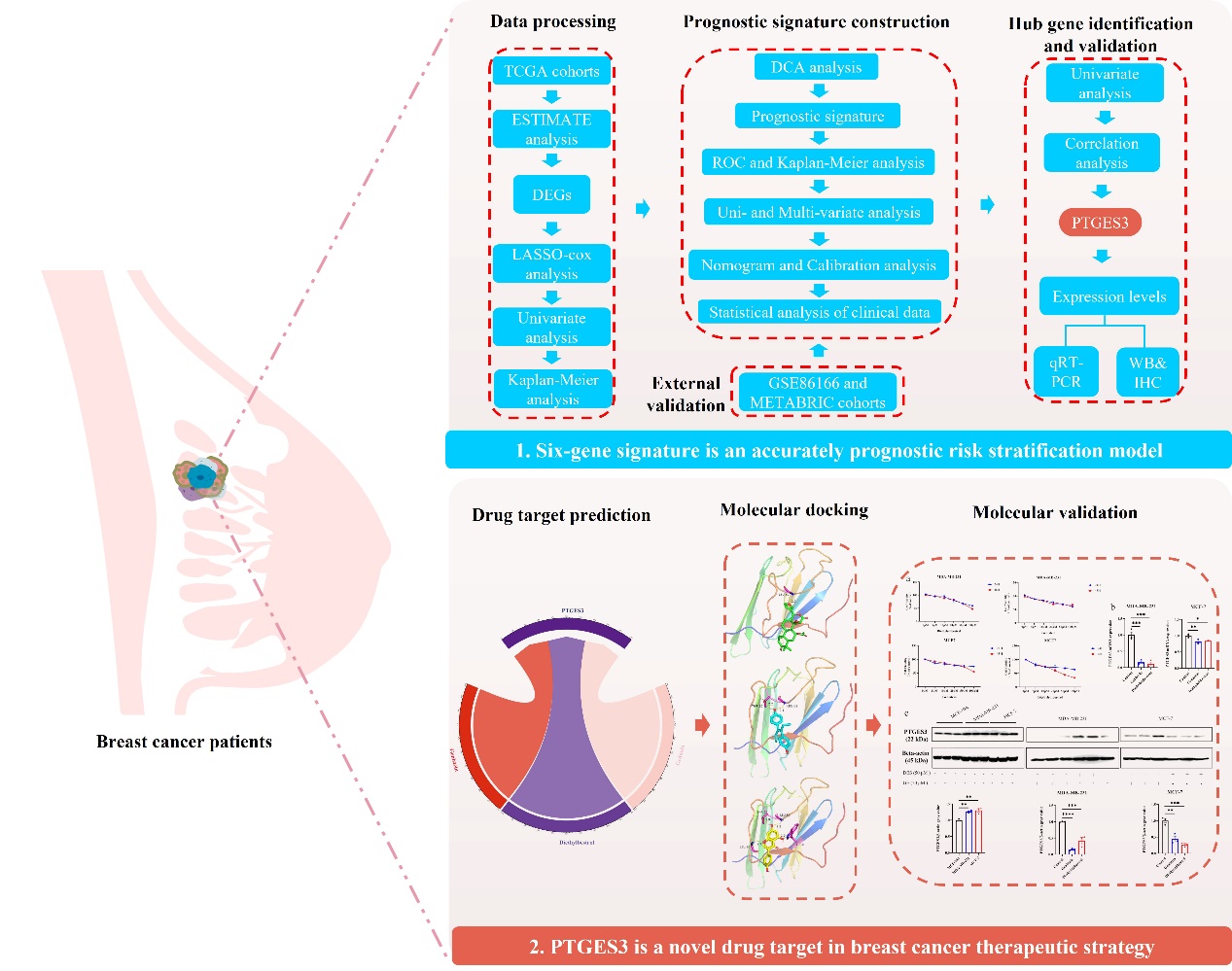


Figure S1 The flow chart for whole process analysis of this study


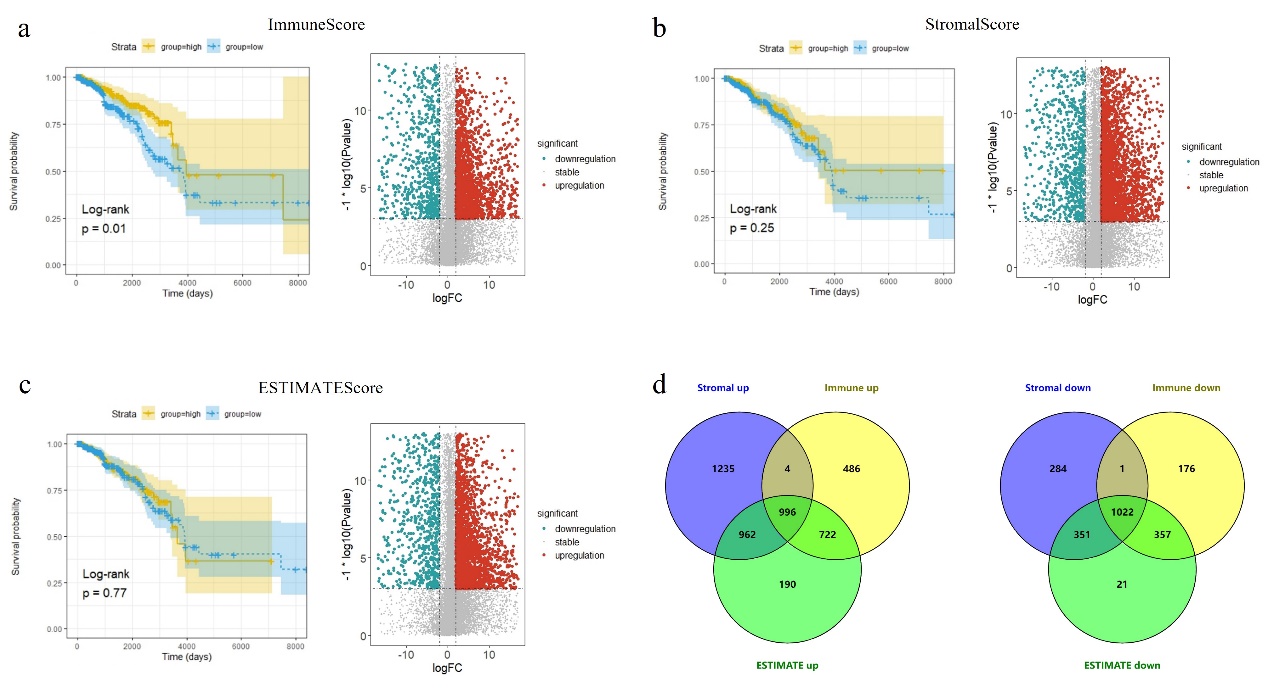


Figure S2 The Kaplan-Meier basing on the ESTIMATE analysis and Venn diagram of the differentially expressed genes (DEGs). The Kaplan-Meier curve and volcano plot basing on (a) immune score, (b) stromal score and (c) ESTIMATE score; (d) Venn diagram of the up-regulation and down-regulation DEGs. The overlapped DEGs are used for further analysis.


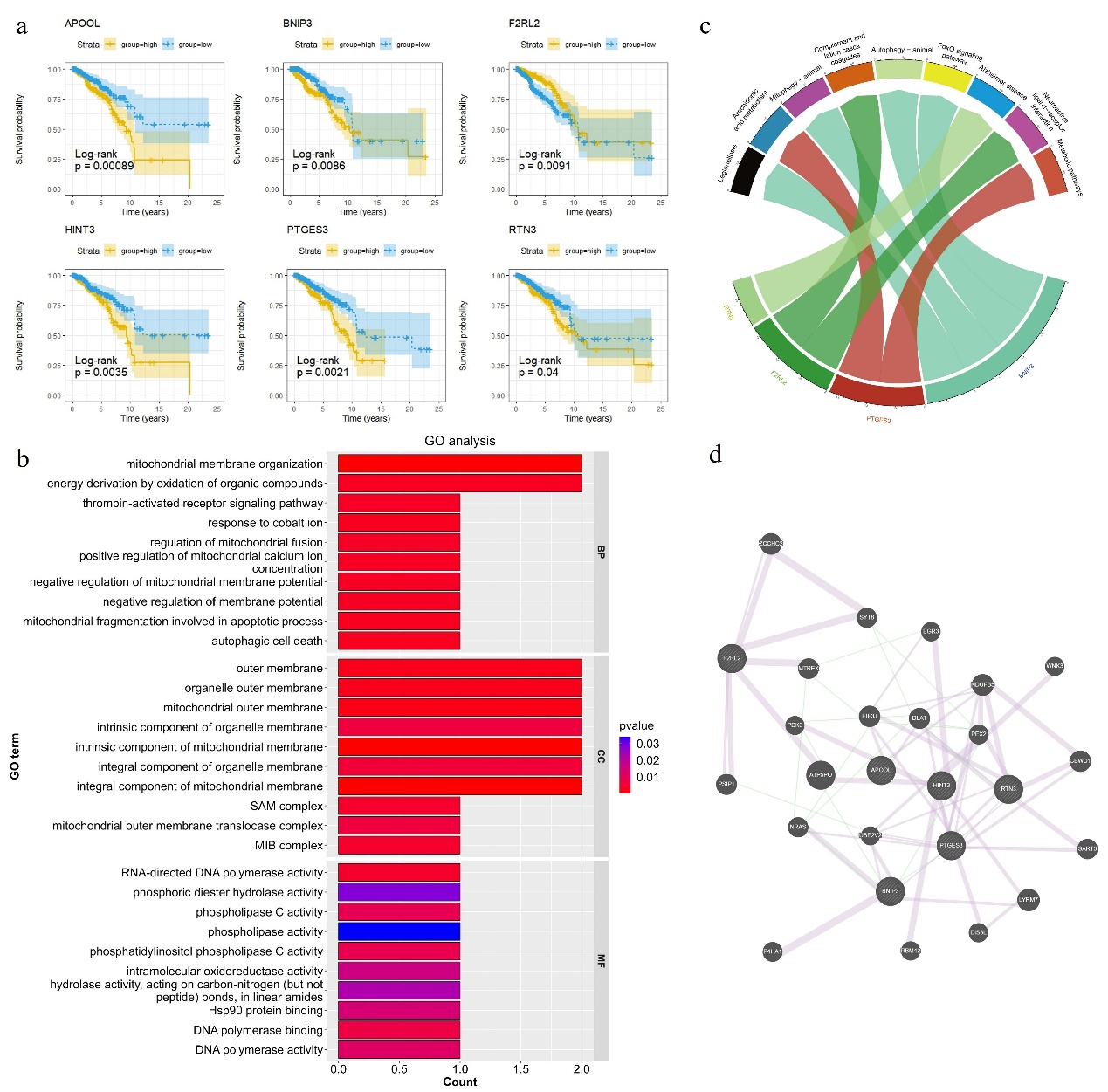


Figure S3 The Kaplan-Meier and functional analysis. (a) Survival analyses according to the optimal cut-off expression value of each gene in the TCGA-BRCA cohort. All *p* < 0.05; (b) GO enrichment analysis; (c) KEGG analysis; (d) Protein-protein interaction analysis of six genes.


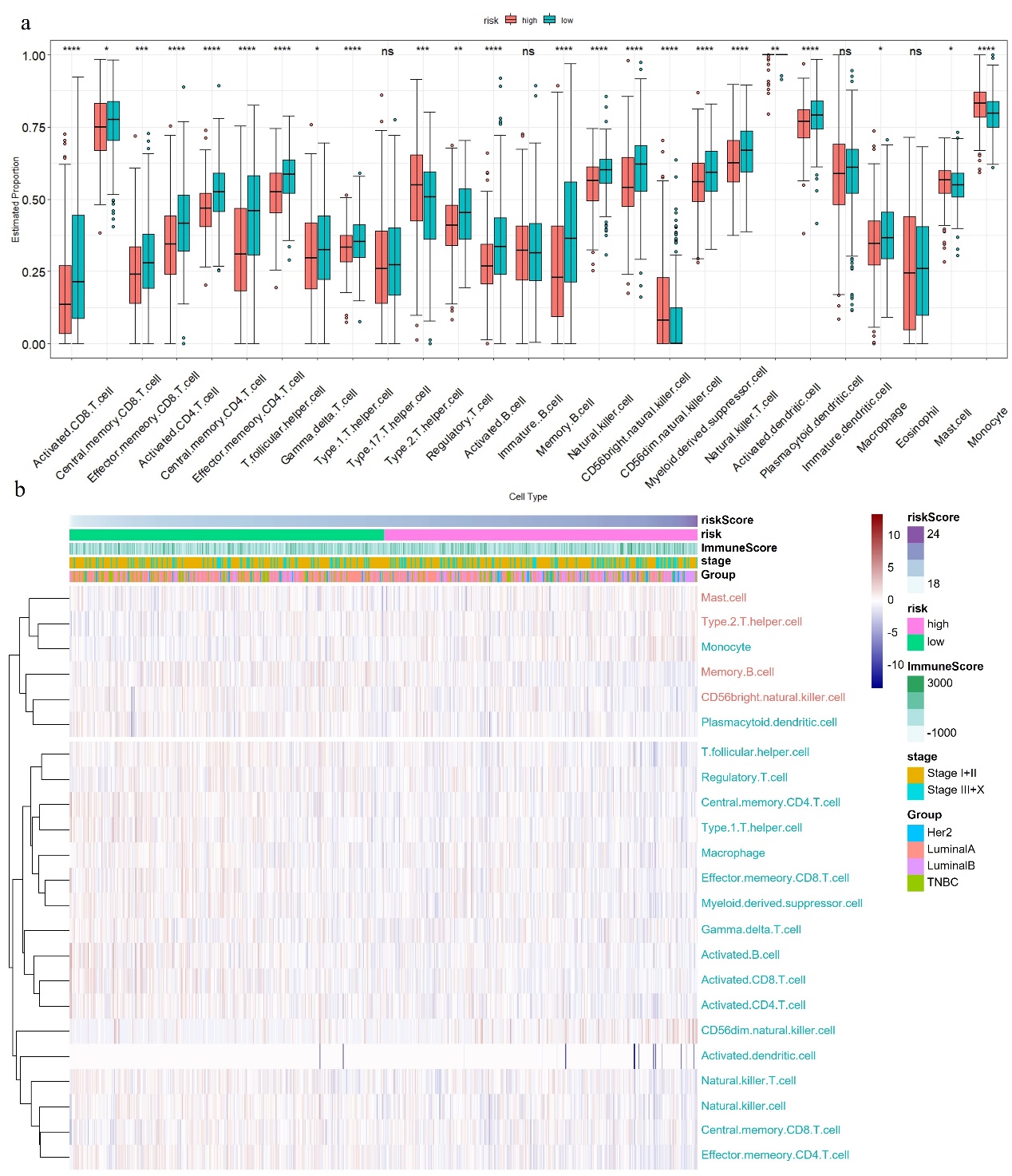


Figure S4 Single sample gene set enrichment (ssGSEA) analysis in TCGA-BRAC cohort. (a) The expression levels of different immune cells in low- and high-risk groups; (b) The distribution of immune cells; red font represents upregulation and blue font represents downregulation; **p* < 0.05, ***p* < 0.01; ****p* < 0.005, *****p* < 0.001.


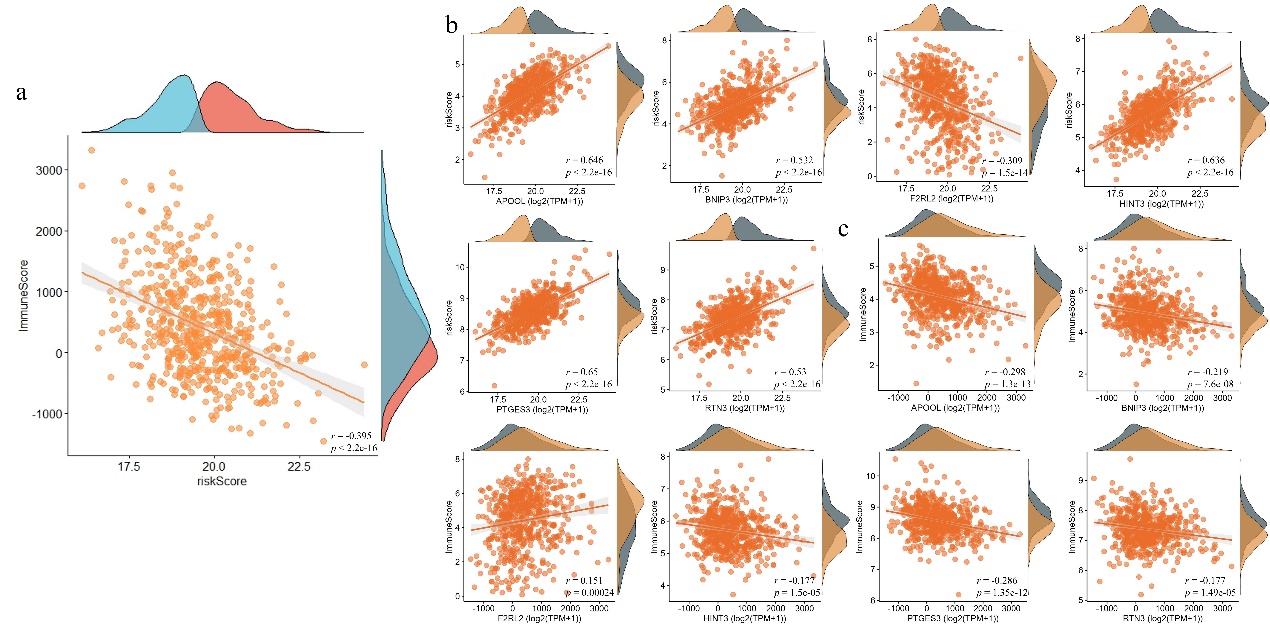


Figure S5 Correlation analysis for the six genes in TCGA-BRAC cohort. (a) The correlation between riskScore and immunScore; (b) The correlation between riskScore and 6 genes; (c) The correlation between immuneScore and six genes.
